# Retrospective Analysis of Monkeypox Infection

**DOI:** 10.3201/eid1404.071044

**Published:** 2008-04

**Authors:** Melissa E. Dubois, Mark K. Slifka

**Affiliations:** * Oregon Health and Science University, Beaverton, Oregon, USA

**Keywords:** Monkeypox, vaccinia, differential diagnosis, serologic diagnostics, research

## Abstract

Serologic cross-reactivity between orthopoxviruses is a substantial barrier to laboratory diagnosis of specific orthopoxvirus infections and epidemiologic characterization of disease outbreaks. Historically, time-consuming and labor-intensive strategies such as cross-adsorbed neutralization assays, immunofluorescence assays, and hemagglutination-inhibition assays have been used to identify orthopoxvirus infections. We used cross-adsorption to develop a simple and quantitative postadsorption ELISA for distinguishing between monkeypox and vaccinia infections. Despite the difficulty of diagnosing clinically inapparent monkeypox in previously vaccinated persons, this technique exhibited 100% sensitivity and 100% specificity for identifying clinically overt monkeypox infection irrespective of vaccination history. We also describe a Western blot technique in which up to 3 diagnostic bands may be used to distinguish between vaccinia and monkeypox infection. The techniques described provide independent diagnostic tests suitable for retrospective analysis of monkeypox outbreaks.

## Abstract 2

Tests have been developed and optimized for serologic differentiation between monkeypox- and vaccinia-induced immunity.

Human monkeypox is a zoonotic disease found in remote areas of western and central sub-Saharan Africa and is an important public health issue in these areas (*1*,*2*). Clinical symptoms of monkeypox can be similar to those of chickenpox (caused by varicella-zoster virus), and these symptoms can cause difficulties in diagnosing cases on the basis of clinical symptoms alone (*2**,**3*). These symptoms can also vary among persons; most American patients in the 2003 monkeypox outbreak had a rash with 1 to >100 skin lesions (*4**–**7*), whereas others may have monkeypox infection without exanthem (*8**,**9*). Moreover, adults with preexisting immunity from childhood smallpox vaccinations may experience milder symptoms (*5**,**6**,**10*) or no symptoms (*6*). Standard clinical algorithms (*11**,**12*) may fail to identify these mild or asymptomatic cases; likewise, they are difficult to confirm by virologic methods because of the absence of skin lesions. Because of the extensive variability in clinical symptoms and common misdiagnosis as chickenpox, developing multiple diagnostic techniques that can be used to identify and confirm monkeypox is crucial.

We describe 2 serologic techniques for diagnosing monkeypox infection. These techniques were based on diagnostic approaches such as radioimmunoassays and neutralization assays that used a preadsorption step to remove or reduce cross-reactive orthopoxvirus antibodies before detection of species-specific antiviral antibodies (*13*–*15*). These basic ELISA and Western blot methods will be useful for orthopoxvirus-specific serosurveys and retrospective analysis of monkeypox outbreaks.

## Materials and Methods

### Participants

Adults previously characterized as having suspected, probable, or confirmed cases of monkeypox during the 2003 Wisconsin monkeypox outbreak provided informed written consent and completed a medical history questionnaire before participation in our previous study (*6*). Monkeypox confirmation was based on the standard case definition and confirmation by the Centers for Disease Control and Prevention (CDC) in Atlanta, Georgia, USA, during the outbreak (*4*) or by subsequent immunologic tests (*6*). Participants were grouped according to vaccination history (Table). Samples from 13 unvaccinated monkeypox-immune persons and 8 vaccinated monkeypox-immune persons were assessed. Of the 8 vaccinated persons, 3 had clinically inapparent cases and were not aware of having been infected with monkeypox because of no noticeable disease symptoms (*6*). However, these are described as laboratory-confirmed cases of monkeypox on the basis of multiple immunologic assays (*6*) (Table). Control participants included recent primary smallpox vaccinees (n = 10), revaccinated persons (n = 10) examined 2–4 months postvaccination, long-term immune smallpox vaccinees examined 20–40 years postvaccination (n = 10), and unvaccinated orthopoxvirus-naive persons (n = 12). Heparinized blood was centrifuged over Histopaque-1077 (Sigma, St. Louis, MO, USA), and plasma was collected and stored at –80°C. Studies involving human participants were reviewed and approved by the Institutional Review Board for Oregon Health and Science University.

**Table:** Comparison of monkeypox-specific diagnostic tests*

Patient no.	Serologic techniques		Cellular techniques	Direct viral detection	Postadsorption ELISA	Postadsorption Western blot†		
	Paired IgG	Peptide ELISA				39 kDa	124 kDa	148 kDa
Monkeypox								
447	C	C	C	C	C	+	+	+
452	C	C	C	C	C	+	+	+
453	C	C	C	P/S	C	+	+	+
461	C	C	C	NA	C	+		+
462	C	C	C	C	C	+		
481	C	C	C	NA	C	+	+	
482	C	C	C	NA	C	+	+	
484	C	C	C	NA	C	+		
489	C	C	C	NA	C	+	+	
473	C	C	C	NA	C	+	+	
519	C	C	C	C	C	+	+	
520	C	C	C	C	C	+	+	
557	ND	ND	ND	NA	C	+	+	+
Vaccinia–monkeypox								
446‡	C	C	C	ND	U			
449‡	C	C	U	ND	U	+		
450	C	C	C	P/S	C	+		
451	C	C	C	C	C	+		
454	C	C	C	P/S	C	+	+	+
455‡	C	C	C	ND	U	+		
463	C	C	C	C	C	+		
500	C	C	C	ND	C			+

*These studies are based on persons who were infected with monkeypox during the 2003 outbreak in Wisconsin and who were tested by independent laboratories by using serologic (*6*), cellular (*6*), and virologic (*4*) techniques. Serologic techniques included identification of monkeypox peptide-specific immunoglobulin (Ig) G by peptide ELISA and kinetic analysis of IgG to orthopoxvirus in paired plasma samples (2–4 mo and 1 y postinfection) by endpoint ELISA. Orthopoxvirus-specific CD8+ T cells were measured by using intracellular cytokine staining analysis. Plasma samples were obtained 2–4 mo postinfection, except for patients 473 and 500 (6 mo), 519 and 520 (12 mo), and 557 (30 mo). Virologic confirmation was obtained by viral culture, PCR, electron microscopy, and immunohistochemical analysis of tissue samples (*4*). C, confirmed; P/S, probable/suspected; NA, not available; ND, not done; U, unconfirmed. †A + indicates that at least 5/6 analysts scored the band of interest as positive. ‡Clinically inapparent monkeypox infection in previously vaccinated persons as determined by cellular and serologic retrospective diagnostic techniques (*6*).

### Viruses and Cells

Monkeypox virus (Zaire strain) and vaccinia virus (Western Reserve strain) were grown in BSC40 cells by using a multiplicity of infection of 0.1 and harvested at 48 h postinfection. Cells were lysed by 3 freeze/thaw cycles in 10 mmol/L Tris, pH 8.0, and used as a virus lysate for preadsorption in ELISA and Western blots. Where indicated, monkeypox and vaccinia viruses were also purified by ultracentrifugation through 36% sucrose at 40,000 × *g* for 80 min, followed by band extraction after 25%–40% sucrose gradient sedimentation (33,000 × *g* for 40 min). Protein concentration was measured by the modified Lowry assay (Pierce, Rockford, IL, USA). Purified virus was inactivated for 2 h at 56°C; viral lysates were inactivated with 3% H_2_O_2_ at room temperature for 2 h. Uninfected BSC40 cell lysate was treated similarly to the infected cells described above.

### Postadsorption ELISA

High protein-binding ELISA plates (Corning-Costar, Corning, NY, USA) were coated with an optimized concentration of H_2_O_2_-inactived monkeypox-infected BSC40 cell lysate. Plasma samples were preadsorbed with equivalent (6 ×10^8^ PFU/mL) concentrations of H_2_O_2_-inactived monkeypox or vaccinia whole-cell lysate at a 1:30 dilution (5 μL plasma in 145 μL viral lysate) for 30 min at 37°C. Nonadsorbed samples were similarly treated with ELISA blocking buffer (phosphate-buffered saline containing 5% nonfat dry milk and 0.05% Tween 20). Samples were then added directly to ELISA plates, serially diluted in blocking buffer, and incubated at room temperature for 1 h. As a precaution against human blood-borne pathogens, samples were then treated with 3% H_2_O_2_ (final concentration) for an additional 30 min. After washing, horseradish peroxidase–conjugated mouse antihuman immunoglobulin (Ig) G monoclonal antibody (clone G18–145; BD Pharmingen, San Diego, CA, USA) was added to the wells. Plates were washed after 1 h and detection reagents were added. Substrate was prepared (*o*-phenylenediamine; Sigma-Aldrich, St. Louis, MO, USA) and diluted to a concentration of 0.4 mg/mL in 0.05 M citrate, pH 5.0, and H_2_O_2_ was added (final concentration of 0.01%). Color was developed for at least 20 min before the reaction was stopped by adding 1 M HCl; plates were read at 490 nm.

Antibody titers were determined by log-log transformation of the linear portion of the dilution curve with 0.1 optical density units used as the endpoint, and transformation was performed on final values (*6*). For an Excel (Microsoft, Redmond, WA, USA) file containing a template of these calculations, please contact the corresponding author, or see the example given in the online Technical Appendix (available from www.cdc.gov/EID/content/14/4/592-Techapp.pdf).

### Differential Western Blot

Western blot procedures were performed with the following modifications. Two micrograms of gradient-purified monkeypox or vaccinia virus was separated by 4%–20% Tris-glycine gradient sodium dodecyl sulfate–polyacrylamide electrophoresis (SDS-PAGE) (Invitrogen, Carlsbad, CA, USA) under reducing conditions. Equivalent protein loading was confirmed on representative gels by staining with GelCode Blue (Pierce).

Proteins were electrophoretically transferred to polyvinylidene difluoride membranes (Pierce), and membrane strips with 3 lanes containing a molecular mass standard (SeeBlue Plus 2; Invitrogen), monkeypox, and vaccinia were blocked with phosphate-buffered saline containing 1% Tween 20 and 5% nonfat dry milk. Plasma was diluted 1:20 in uninfected cell lysate or H_2_O_2_-inactived vaccinia lysate (adjusted to a concentration of 2.5 mg/mL total protein) for 30 min at 37°C. Adsorbed plasma was adjusted to a 1:10,000 dilution in 10 mL of blocking buffer and incubated with membranes overnight in 50-mL conical tubes at 4°C with rocking. After 3 washes in blocking buffer, reactive bands were identified with horseradish peroxidase–conjugated goat antihuman IgG (γ chain specific; Jackson ImmunoResearch, West Grove, PA, USA) by using chemiluminescent detection (SuperSignal West Dura Substrate; Pierce). Plasma from the same monkeypox patient was used as a positive control in each experiment to identify the position of diagnostic bands. Blots were exposed to x-ray film until diagnostic bands were clearly visible, and other films were then overexposed to ensure that any low-intensity bands were given ample opportunity to appear. Plasma from some orthopoxvirus-naive persons did not react with any monkeypox or vaccinia protein bands. In these instances, films were exposed 10× longer than the last readable positive control exposure before a negative result was recorded. Films were scanned and the positions of diagnostic bands were indicated on the basis of the positive control. Analysts scored the vaccinia-preadsorbed Western blots for the 39-kDa, 124-kDa, and 148-kDa diagnostic bands as present only in the monkeypox lane, absent from the monkeypox lane, present in the monkeypox and vaccinia lanes (i.e., experimental equivocal), or technical equivocal caused by nonspecific background. Immunoreactive bands deemed experimental equivocal were counted against the sensitivity or specificity of the assay. Blots containing technical equivocal data were repeated, and analysts rescored bands that were unreadable in the initial screen before determining final sensitivity and specificity.

## Results

### Postadsorption ELISA

Orthopoxviruses have highly conserved genomes (*16*), which results in high levels of antibody cross-reactivity. However, viruses such as monkeypox also contain genes that are absent, mutated, or truncated in vaccinia; these gene products can be used to distinguish between monkeypox and vaccinia infections (*6**,**17*–*21*). The concentration of viral antigen used in the preadsorption step of the ELISA was determined by using high-titer plasma from 2 recently vaccinated vaccinia-immune persons obtained at the peak (day 21) of the anamnestic response with ELISA titers that were ≈10-fold higher than the highest convalescent-phase samples used in the rest of the experiments. Two of the highest titer samples from monkeypox-immune persons (obtained at 2 months postinfection) were also used in these preliminary studies online Technical Appendix (available from www.cdc.gov/EID/content/14/4/592-Techapp.pdf). These samples provided a rigorous test of homologous and heterologous antigens to deplete antiviral antibodies before performing the ELISA. Titration of virus antigen indicated that lysates normalized to contain 6 × 10^8^ PFU equivalents/mL were best for differentiating between vaccinia and monkeypox infections.

**Figure 1:**
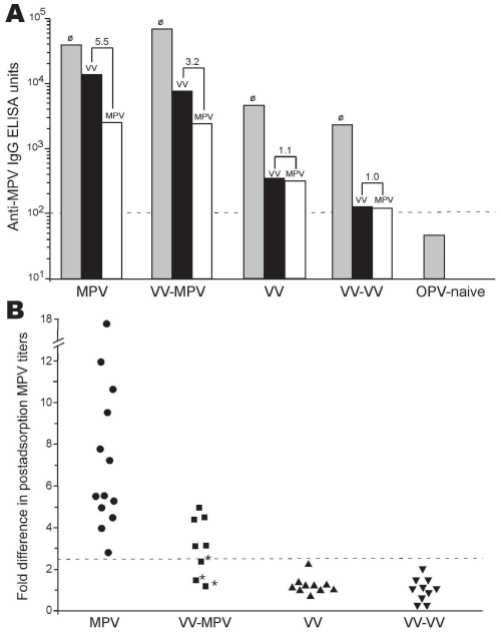
Diagnosis of monkeypox by postadsorption ELISA. Plasma samples were obtained from monkeypox-immune persons (2–30 months postinfection), vaccinia-immune persons (2–4 months postinfection), or uninfected orthopoxvirus-naive persons and tested on ELISA plates coated with inactivated monkeypox antigen. A) A representative monkeypox-specific ELISA with plasma samples from an unvaccinated monkeypox-infected person (MPV), a previously vaccinated (i.e., vaccinia-immune) monkeypox-infected person (VV-MPV), a vaccinia-immune person (VV), a vaccinia-immune person who was revaccinated with vaccinia (VV-VV), and an uninfected orthopoxvirus-naive person (OPV-naive). Plasma was not preadsorbed (∅, gray bars), preadsorbed with inactivated vaccinia antigen (black bars), or preadsorbed with inactivated monkeypox antigen (white bars) before ELISA on monkeypox-coated plates. Numbers above bars refer to differences in postadsorption MPV ELISA titers after adsorption with vaccinia antigen compared with adsorption with monkeypox antigen. Plasma from 1 orthopoxvirus-naive person (representative of n = 12) was not preadsorbed with viral antigen because it was seronegative (<100 ELISA units) and below our detection limit (dashed horizontal line). B) Plasma samples from monkeypox-infected persons (•, n = 13), vaccinia-immune monkeypox-infected persons (■, n = 8), vaccinia-immune persons (▲, n = 10), and revaccinated vaccinia-immune persons (▼, n = 10) were tested by postadsorption ELISA. Data show fold-differences of monkeypox antibody titers after adsorption with vaccinia antigen compared with adsorption with monkeypox antigen. Dashed horizontal line indicates a diagnostic cutoff indicative of a positive result, which was determined as a postadsorption difference score of >2.5. *Denotes results of plasma samples obtained from persons with clinically inapparent monkeypox infection.

Orthopoxvirus-naive persons or persons infected with monkeypox or vaccinia were then tested to establish the diagnostic validity of this approach by using monkeypox-coated ELISA plates (Figure 1, **panel A**). If plasma from a representative monkeypox-immune person was preincubated with vaccinia antigen, virus-specific antibody titers decreased (from 39,904 ELISA units [EU] to 13,912 EU). However, if the sample was preincubated with an equivalent amount of monkeypox antigen before performing the ELISA, monkeypox antibody titers were further decreased, to 2,542 EU. For the data of the monkeypox-immune person shown in Figure 1, **panel A**, there was a 5.5-fold difference (13,912 EU divided by 2,542 EU) in the ability of monkeypox antigen to reduce monkeypox-specific antibody levels than vaccinia antigen. For persons who were vaccinia immune but still had contracted clinically apparent monkeypox disease, the difference in postadsorption ELISA titers was typically smaller (3.2-fold difference in the example shown in Figure 1, **panel A**), but the difference still demonstrated a clear distinction between preadsorption with monkeypox or vaccinia antigens. If a person had been infected with only vaccinia or revaccinated with vaccinia, there was essentially no difference in the ability of monkeypox antigen to deplete antiviral antibodies than an equivalent amount of vaccinia antigen on a monkeypox-coated ELISA plate. In contrast, orthopoxvirus-naive persons were seronegative by ELISA and can be easily distinguished from monkeypox-immune or vaccinia-immune persons because they score below the limits of detection by ELISA (<100 EU) without any preadsorption steps required.

To determine whether the difference in postadsorption ELISA results could be used effectively to distinguish between vaccinia and monkeypox infections, we plotted the results from primary monkeypox-immune persons, previously vaccinated monkeypox immune persons, primary vaccinia-immune persons, and revaccinated vaccinia-immune persons (Figure 1, **panel B**). We established a diagnostic cutoff of 2.5 by plotting the difference in postadsorption ELISA titers for each group. To establish the diagnostic cutoff, we used persons with laboratory-confirmed cases of primary monkeypox (*6*) as positive controls and vaccinia-immune persons with no evidence of monkeypox infection as negative controls (Figure 1, **panel B**). The 2.5-fold difference in postadsorption ELISA titers provided the best balance of high sensitivity (100% positive for primary monkeypox) and high specificity (0% positive for vaccinia immune). Other diagnostic cutoff values resulted in decreased specificity or sensitivity when these 2 divergent groups of positive and negative controls were compared. For example, a diagnostic cutoff of 3.0 reduced the sensitivity of the assay to 93% (12/13) for primary monkeypox but did not affect the specificity (100%). Conversely, a diagnostic cutoff of 2.0 did not affect sensitivity (100%) but decreased specificity from 100% to 90% (i.e., 18/20 vaccinia-immune persons were positive). Vaccinia-immune monkeypox patients typically had lower differences in postadsorption ELISA titers. The lower difference suggests that preexisting immunity to vaccinia may have resulted in a lower induction of monkeypox species-specific antibody responses and is consistent with the results of our previous study (*6*).

Using the established 2.5-fold diagnostic cutoff, we achieved 100% (13/13) sensitivity for detecting primary monkeypox infection and 63% (5/8) sensitivity for detecting monkeypox infection in vaccinia-immune persons in which monkeypox was a heterologous orthopoxvirus infection. Of the 8 vaccinia-immune persons with monkeypox, 3 were clinically asymptomatic but were previously identified by serologic and cellular techniques (*6*). However, these cases are more difficult to diagnose on the basis of serologic analysis only (*6*) and could not be identified by the postadsorption ELISA (Figure 1, **panel B**; Table). However, if only persons with clinically overt monkeypox infection were included in the analysis, 100% (5/5) of secondary monkeypox infections were identified by this assay (Figure 1, **panel B**; Table). We also observed 100% specificity; 20/20 vaccinia-immune controls (10 primary and 10 booster smallpox vaccinations) and 12/12 orthopoxvirus-naive persons were negative by this postadsorption ELISA. We report 86% (18/21) overall sensitivity for detecting monkeypox, 100% (18/18) sensitivity for confirming clinically overt monkeypox, and 100% (32/32) specificity with this approach.

### Differential Western Blot

**Figure 2:**
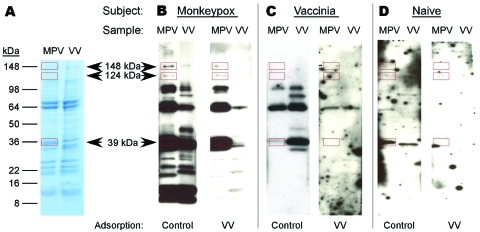
Development of a monkeypox (MPV)–specific diagnostic assay using Western blot analysis. Adsorption of cross-reactive orthopoxvirus antibodies with vaccinia antigen before Western blot analysis provided easier identification of monkeypox-specific bands. A) Two micrograms of sucrose gradient–purified monkeypox virus or vaccinia virus (VV) were separated by sodium dodecyl sulfate–polyacrylamide gel electrophoresis (4%–20% gels) and stained with GelCode Blue (Pierce, Rockford, IL, USA) to compare banding patterns and confirm equivalent protein loading. Proteins were electrophoretically transferred to polyvinylidene difluoride membranes and probed with plasma from B) a monkeypox-immune person, C) a vaccinia-immune person, or D) an orthopoxvirus-naive person after adsorption of plasma with control antigen (uninfected H_2_O_2_-treated BSC40 cell lysate) or vaccinia antigen (H_2_O_2_-inactived vaccinia-infected BSC40 cell lysate). Immunoreactive bands were detected with peroxidase-conjugated antihuman immunoglobulin G plus chemiluminescent substrate and exposed to x-ray film. Arrows indicate location of diagnostic bands with apparent molecular masses of 148, 124, and 39 kDa. Rectangles indicate locations of diagnostic bands.

To more easily recognize uniquely reactive monkeypox-specific bands by Western blot and simplify interpretation of this serologic diagnostic technique, we added an adsorption step to reduce cross-reactive antibodies to orthopoxvirus. Separation of 2 μg of purified vaccinia and monkeypox by 4%–20% gradient SDS-PAGE resulted in good separation of protein bands across a broad spectrum of molecular masses (Figure 2, **panel A**) and effective transfer of separated proteins to the polyvinylidene difluoride membrane. Protein-banding patterns of monkeypox virus and vaccinia virus are similar, but a protein band of ≈39 kDa is visible in the monkeypox lane of the GelCode Blue-stained SDS-PAGE that is missing from the vaccinia lane. Incubation of immune plasma in a 20-fold volume excess of virus antigen (1 μL plasma plus 19 μL vaccinia-infected cell lysate at 6 × 10^8^ PFU equivalents/mL) before performing the Western blot effectively reduced the intensity and detection of cross-reactive bands (Figure 2, **panel B**).

Diagnostic bands with apparent molecular masses of 148, 124, and 39 kDa were identified when monkeypox-immune plasma was used, but were not observed after Western blot analysis with plasma samples from a representative vaccinia-immune person (Figure 2, **panel C**) or an orthopoxvirus-naive person (Figure 2 , **panel D**). Because orthopoxvirus-naive persons were seronegative by ELISA (Figure 1, **panel A**), the Western blot example shown in Figure 2, **panel D**, required a 1-hour exposure to identify any faint bands after vaccinia antigen adsorption. In contrast, samples from monkeypox-immune and vaccinia-immune persons were exposed to film for 5 s to 2 min for optimal identification of virus-specific banding patterns. We did not observe a correlation between viral protein levels determined by SDS-PAGE (Figure 2, **panel A**) and immunodominance by Western blot analysis (Figure 2, **panel B**).

**Figure 3:**
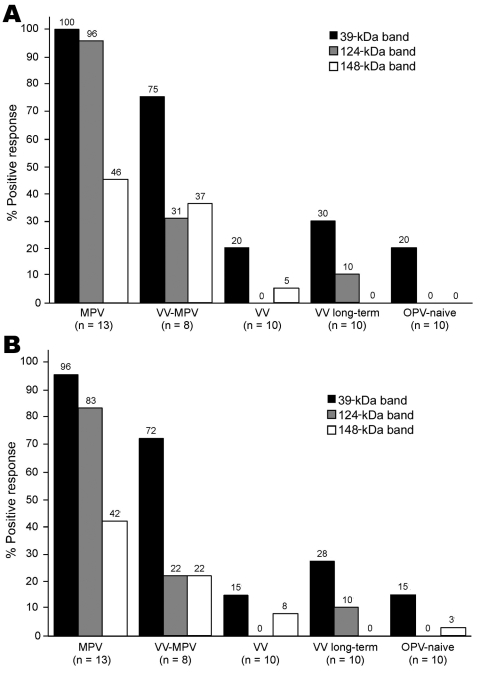
Diagnosis of monkeypox infection by Western blot analysis. Plasma samples from unvaccinated monkeypox-infected (2–30 months postinfection) (MPV), vaccinia-immune monkeypox-infected (2–6 months postinfection) (VV-MPV), primary vaccinia-immune (2–4 months postimmunization) (VV), long-term vaccinia-immune (>20 years postimmunization) (VV long-term), and orthopoxvirus-naive (OPV) persons were analyzed by Western blot after adsorption with vaccinia-infected BSC40 cell lysate to reduce cross-reactive antibodies as described in Figure 2. Immunoreactivity to diagnostic protein bands of ≈39 kDa, 124 kDa, and 148 kDa was assessed by A) unblinded analysts with knowledge of subject medical history (n = 2) and B) blinded analysts who did not have knowledge of subject medical history (n = 4). Findings of each analyst were averaged for each person and percentages shown represent a composite of all data points.

Unblinded analysis of the Western blots was first performed by 2 independent analysts who had access to patients’ medical histories (Figure 3, **panel A**). Reactivity to the 39-kDa band resulted in 100% sensitivity for identifying primary monkeypox infection and 75% sensitivity for identifying secondary monkeypox infection. However, 20%–30% of the negative control samples (vaccinia primary, vaccinia long-term, and orthopoxvirus naive) also reacted with this band, which resulted in a specificity of only 70%–80% (76% overall specificity). The 124-kDa band showed 96% sensitivity for identifying primary monkeypox infection but only 31% sensitivity for identifying secondary monkeypox infection. However, this protein was more specific than the 39-kDa protein; 0%–10% of negative controls showed reactivity (97% overall specificity). The 148-kDa band showed low diagnostic sensitivity; only 37%–46% of plasma samples from monkeypox-immune persons reacted with it in Western blot analysis. Conversely, the 148-kDa band showed high specificity with 0%–5% of negative control plasma that were positive (i.e., overall specificity >97%). Other bands were also identified as potential diagnostic indicators of monkeypox infection, such as the 98-kDa and 18-kDa bands (Figure 2, **panels B** and **C**; online Technical Appendix, http://www.cdc.gov/EID/content/14/4/592-Techapp.pdf). However, further analysis showed that the 98-kDa band had low diagnostic potential (48% sensitivity and 72% specificity), as did the 18-kDa band shown in Figure 2, **panel B** (60% sensitivity and 66% specificity).

Blinded analysts (n = 4) with no knowledge of infection history were asked to score Western blots to determine the feasibility of this approach under conditions in which background clinical information may not be available (Figure 3, **panel B**). Unblinded analysts reported somewhat higher sensitivity for each of the diagnostic bands and identified the same or slightly lower specificity when interpreting Western blots with vaccinia-immune or orthopoxvirus-naive plasma samples, but the overall results from blinded and unblinded analysis were similar.

## Discussion

Historically, several serologic techniques have been used to identify orthopoxvirus infections, including hemagglutination-inhibition (*13*), gel precipitation (*17**,**22**,**23*), complement fixation (*22*), cross-neutralization (*14*), immunofluorescence (*24**,**25*), Western blot (*26*,*27*), radioimmunoassay (*13*,*15*), and ELISA (*28**,**29*). In previous studies, we used a peptide-based ELISA with peptide sequences from the monkeypox B21R gene to differentially diagnose monkeypox infection (*6*). This approach is highly sensitive and specific for diagnosing monkeypox at 2–4 months postinfection. However, it is yet unknown how quickly peptide-specific antibody responses are mounted or how long they are maintained. Because multiple independent diagnostic techniques should be available for detecting virulent orthopoxvirus infections, we have developed the 2 new assays.

Detection of virus-specific IgM by ELISA is often considered the most useful serologic technique for confirming a recent infection. An IgM capture ELISA for diagnosing monkeypox (*29*) was developed by using vaccinia virus as a surrogate antigen for monkeypox, and the investigators reported 100% specificity and 92% sensitivity. Per CDC monkeypox case definition, these cases had been confirmed by direct viral detection including PCR, electron microscopy, virus isolation, or immunohistochemistry. However, the reported specificity and sensitivity were based on only a subset of samples obtained 5–77 days after onset of rash. Inclusion of samples beyond this range resulted in additional false-negative results and a concomitant decrease in sensitivity. A further limitation of this study is that only virologically confirmed monkeypox cases were included; persons with mild symptoms or vaccine-mediated subclinical infection were not examined.

A more recent study identified 3 unvaccinated contacts who were negative by IgM ELISA but positive by IgG ELISA despite no reported disease symptoms (*10*). If these contacts contracted monkeypox infections, then this would contradict our previous findings in which unvaccinated monkeypox patients showed at least some, if not all, disease symptoms associated with monkeypox (*6**,**8*). It is difficult to directly compare our results with these other reports (*10**,**29*) because different subsets of monkeypox patients were examined, different time points were analyzed, and different case definitions were used. For instance, the CDC case definition is based on a combination of clinical, epidemiologic, and laboratory criteria (www.cdc.gov/ncidod/monkeypox/casedefinition.htm); on the basis of these criteria, monkeypox infection can only be confirmed if virus is detected.

We used epidemiologic criteria (i.e., direct or indirect exposure to monkeypox-infected animals or humans) in addition to laboratory criteria with high sensitivity and specificity (*6*). This approach led to identification of persons in whom monkeypox exposure and disease symptoms occurred, but only immunologic and not virologic laboratory analysis could be performed. Moreover, we identified 3 previously vaccinated persons who were exposed to monkeypox and showed immunologic evidence of infection (*6*). However, because they did not show clinical disease symptoms, they would not meet CDC case definition for monkeypox.

Depletion of cross-reactive antibodies by preadsorption before ELISA enabled the identification of clinically apparent monkeypox infection with 100% sensitivity and 100% specificity. However, 1 limitation was that we were unable to identify clinically inapparent monkeypox infection in previously vaccinated persons (Figure 1, Table). These persons were not tested by virologic methods but were confirmed by immunologic techniques such as early versus late analysis for antibodies to orthopoxvirus, monkeypox B21R peptide ELISA, or orthopoxvirus-specific T-cell analysis (*6*). In our previous study (*6*) and this study, persons with high antiviral immunity from smallpox vaccinations were more likely to mount anamnestic responses to cross-reactive vaccinia epitopes rather than to monkeypox. This may be caused by more limited monkeypox replication, which resulted in fewer disease symptoms (*6*) and less antigenic stimulation of antibody responses to novel virus epitopes. Despite the difficulties involved with serologic diagnosis of clinically asymptomatic cases of monkeypox, the postadsorption ELISA described here provides a robust method for distinguishing between vaccinia and clinically overt monkeypox infections.

Our modified Western blot technique provides an additional independent test for laboratory confirmation of orthopoxvirus infection. It has only modest value as a stand-alone diagnostic test because of <90% sensitivity and specificity overall, but may be useful for confirming cases of monkeypox identified by other virologic or serologic approaches. Relative to standard Western blot approaches (*26*,*27*), preadsorption of plasma with vaccinia antigens decreased the intensity and detection of cross-reactive proteins and enabled easier identification of 3 diagnostic protein bands (39, 124, and 148 kDa). In this assay, 95% (20/21) of monkeypox-immune participants (including 2/3 who had clinically inapparent cases) reacted with at least 1 diagnostic protein band. A cluster of 33 kDa- to 42 kDa-vaccinia proteins was previously described as immunoreactive with pooled human vaccinia immune globulin (*27*). This cluster was also evident in the current study (Figure 2, **panels B** and **C**), although detection and intensity of bands within this cluster varied among persons. The 39-kDa monkeypox diagnostic band was readily distinguishable from vaccinia-specific proteins. This band may represent a gene product that is unique to monkeypox, or alternatively it may be common to both viruses but modified in a manner that results in distinct migration characteristics. The immunoreactive protein bands identified were effective in differentiating between monkeypox and vaccinia infection and may prove useful for identifying other virulent orthopoxviruses. Preliminary studies indicate that 5 (83%) of 6 smallpox survivors can be retrospectively diagnosed by using this technique.

Serologic diagnostic techniques provide a broad window of detection relative to direct virus detection, which is limited to the period of active infection/virus replication. Only 37 of >72 suspected or probable cases of monkeypox were confirmed by direct virologic methods during the US outbreak (www.cdc.gov/od/oc/media/mpv/cases.htm) (*6*). The US outbreak provided the first identification of human monkeypox outside Africa and showed the importance of monkeypox and other geographically limited zoonoses in an increasingly connected global community. Better diagnostics will help in measuring the effect of monkeypox in disease-endemic areas and will be important in effective outbreak detection during accidental or intentional release of virulent orthopoxviruses. We have described 2 independent methods for serologic confirmation of monkeypox infection that will be useful for these purposes.

## Supplementary Material

Technical Appendix
