# Supplementary material for: Retrospective Analysis of Monkeypox Infection
Source: Emerg Infect Dis. 2008 Apr;14(4):592–9. doi: 10.3201/eid1404.071044 (PMC2570942; doi:10.3201/eid1404.071044)
Supplement: Technical Appendix [file 07-1044_Techapp.pdf]

Technical Appendix

ELISA Data Analysis Example

Plate 1: 7/7/07

Raw data is imported as a text file from the platereader software.

RAW DATA

	1	2	3	4	5	6	7	8	9	10	11	12
	MPV Immune Subject			VV Immune Subject			Naïve Subject					
Dilution:	MPV adsorbed	VV adsorbed	Unadsorbed	MPV adsorbed	VV adsorbed	Unadsorbed	MPV adsorbed	VV adsorbed	Unadsorbed			
30	0.754	2.708	4.000	0.398	0.436	4.000	0.140	0.181	0.121			
90	0.205	0.855	2.496	0.187	0.179	1.633	0.070	0.088	0.070			
270	0.139	0.418	1.274	0.123	0.122	0.651	0.060	0.064	0.050			
810	0.099	0.210	0.557	0.077	0.084	0.252	0.050	0.059	0.045			
2430	0.073	0.111	0.229	0.070	0.064	0.146	0.017	0.020	0.015			
7290	0.056	0.073	0.119	0.072	0.086	0.092	0.006	0.007	0.005			
21870	0.051	0.058	0.066	0.059	0.091	0.087	0.002	0.002	0.002			
Blank	0.046	0.047	0.050	0.044	0.045	0.048	0.050	0.052	0.051	0.050	0.052	0.051

Average Blank:
AVERAGE(D15:O15)

0.049 The average blank is calculated from 12 wells.

Adjusted OD values are calculated by subtracting the Average Blank OD from each raw data value.

Values between OD 0.05 and 1.5 (those points in the linear portion of the dilution curve) are selected to calculate the final titer.

BLANK SUBTRACTED
Raw OD - D18
Select values 1.5 > x > 0.05 (yellow)

	MPV Immune Subject			VV Immune Subject			Naïve Subject		
Dilution:	MPV adsorbed	VV adsorbed	Unadsorbed	MPV adsorbed	VV adsorbed	Unadsorbed	MPV adsorbed	VV adsorbed	Unadsorbed
30	0.705	2.659	3.951	0.349	0.388	3.951	0.091	0.132	0.072
90	0.156	0.806	2.447	0.138	0.130	1.584	0.021	0.039	0.021
270	0.090	0.369	1.225	0.074	0.074	0.602	0.011	0.015	0.001
810	0.050	0.161	0.508	0.028	0.035	0.203	0.001	0.010	-0.004
2430	0.025	0.062	0.180	0.021	0.015	0.097	-0.032	-0.029	-0.034
7290	0.007	0.024	0.070	0.023	0.037	0.043	-0.043	-0.042	-0.044
21870	0.002	0.009	0.017	0.010	0.042	0.039	-0.047	-0.047	-0.047

Values are next log-transformed by calculating the Log10 of the blank-subtracted OD and the dilution values.

LOG BLANK SUBRACTED
LOG10(Blank Subtracted Value)
LOG10(dilution)

	MPV Immune Subject			VV Immune Subject			Naïve Subject		
	MPV adsorbed	VV adsorbed	Unadsorbed	MPV adsorbed	VV adsorbed	Unadsorbed	MPV adsorbed	VV adsorbed	Unadsorbed
1.477	-0.152			-0.458	-0.412		-1.042	-0.880	-1.143
1.954	-0.806	-0.094		-0.861	-0.886				
2.431	-1.047	-0.432	0.088	-1.129	-1.133	-0.220			
2.908	-1.301	-0.794	-0.294			-0.692			
3.386		-1.209	-0.746			-1.013			
3.863			-1.154						
4.340									

Endpoint values are calculated by Excel using the equation of the linear portion of the dilution curve, and transformed to linear values in the final step.

Endpoint titers are visually confirmed by ensuring that the calculated titer is roughly the dilution where blank subtracted OD is expected to equal 0.1 (blue boxes).

		MPV Immune Subject			VV Immune Subject			Naïve Subject		
		MPV adsorbed	VV adsorbed	Unadsorbed	MPV adsorbed	VV adsorbed	Unadsorbed	MPV adsorbed	VV adsorbed	Unadsorbed
Intercept:	INTERCEPT(D45:D51,$C$45:$C$51)	0.868	1.443	2.229	0.560	0.668	1.774			
Slope:	SLOPE(D45:D51,$C$45:$C$51)	-0.77	-0.78	-0.88	-0.70	-0.76	-0.83			
A490:	0.100	0.100	0.100	0.100	0.100	0.100	0.100			
log A490	LOG10(D64)	-1.000	-1.000	-1.000	-1.000	-1.000	-1.000			
log Dilution	(D65-D62)/D63	2.42	3.14	3.69	2.22	2.21	3.34			
Dilution (endpoint titer)	10^(D66)	262	1390	4875	164	160	2186	27	40	22

Figure 1. Illustration of ELISA data analysis. Optical density values at 490 nm (OD_{490}) values were imported directly into Excel (Microsoft, Redmond, WA) from the ELISA microplate reader (VersaMax; Molecular Devices, Sunnyvale, CA, USA). The average OD_{490} of 12 blank wells (Line 18) was then subtracted from the raw OD_{490} obtained for each sample well. These values were transformed to logarithmic scale to create a log-log curve. We use linear OD_{490} values between 1.5 and 0.05 because these typically fall within the linear portion of the curve and are used to determine the endpoint titer. The slope and intercept of the linear portion of the curve were determined by a standard y = mx + b calculation, and the dilution at which OD_{490} = 0.1 was transformed to a linear value to give a final endpoint titer.
